# Supplementary material for: Predicting Lung Function Using Biomarkers in Alpha-1 Antitrypsin Deficiency
Source: Biomedicines. 2023 Jul 15;11(7):2001. doi: 10.3390/biomedicines11072001 (PMC10377580; doi:10.3390/biomedicines11072001)
Supplement: Supplementary file 1 [file biomedicines-11-02001-s001.zip › biomedicines-2475091-supplementary.pdf]

Supplementary material

Table of Contents

Supplementary material 1

Supplementary results 2

Tables 2

Figures 9

Supplementary results

Tables

	ZZ-AATD COPD n=111
**Age, years**	57 [47-65]
**Sex, male**	63 (56.8)
**Smoking**	
Current	2 (1.8)
Ex	82 (74.5)
Never	26 (23.6)
**FEV1, litres**	1.62 [1.17-2.07]
**FEV1 % predicted**	53.8 [35.5-69.6]
**FVC, litres**	3.78 [3.08-4.84]
**FVC % predicted**	98.2 [82.3-112.4]
**FEV1/FVC**	0.43 [0.33-0.53]
**TLCO, mmol/min/kPa**	4.84 [3.78-6.38]
**TLCO % predicted**	64.1 [47.9-77.6]
**mMRC**	
0	7 (10.9)
1	14 (21.9)
2	20 (31.2)
3	15 (23.4)
4	8 (12.5)

Table S1. Characteristics of ZZ AATD-COPD patients
COPD, chronic obstructive pulmonary disease; FEV1, forced expiratory volume in 1 second; FVC, forced vital capacity; mMRC, modified Medical Research Council dyspnoea scale.

	COPD (n=124)		No COPD (n=76)	
	**Median**	**IQR**	**Median**	**IQR**
**CC16** (ng/ml)	23.97	18	27.41	17.36
**CCL18** (ng/ml)	172.55	107.17	124.32	80.48
**SP-D** (ng/ml)	8.05	7.92	6.86	5.43
**CRP** (mg/l)	2.54	5.22	1.07	4.48
**IL-6** (pg/ml)	0.53	0.53	0.52	0.45
**IL-8** (pg/ml)	0.43	2.31	0.74	2.64
**TNF-alpha** (pg/ml)	0.93	1.8	0.93	2.54

Table S2. Raw biomarker values for AATD patients, with and without COPD. CC16, club cell protein 16; CCL18, chemokine ligand 18; SP-D, surfactant protein D; CRP, C-reactive protein; IL-6, interleukin-6; IL-8, interleukin-8; TNF-alpha, tumour necrosis factor alpha.

	FEV1 (n=110) R^2=0.08		TLCO (n=98) R^2=0.22	
	**Beta1 (SE1)**	**P1**	**Beta2 (SE2)**	**P2**
**Age**	-0.008 (0.006)	0.233	-0.076 (0.017)	<0.001
**Male**	0.287 (0.135)	0.036	1.347 (0.371)	<0.001
**Ex-smoker**	-0.543 (0.169)	0.002	-0.254 (0.457)	0.580
**Current smoker**	-0.685 (0.518)	0.189	-0.888 (1.346)	0.511

Table S3. Multivariable model to predict absolute FEV1 or TL_{CO} at baseline in patients with ZZ AATD-COPD (without biomarkers)

FEV1 or TL_{CO} absolute values were modelled using the equation: FEV1 or TL_{CO} ~ Age + Sex + Smoking. The beta coefficients (Beta1 and Beta2), standard error (SE1 and SE2), p-values (P1 and P2), adjusted R^2 (R^2) and number included in each model (n) are presented in the table.

	FEV1 (n=110) R^2=0.20		TLCO (n=98) R^2=0.07	
	**Beta1 (SE1)**	**P1**	**Beta2 (SE2)**	**P2**
**Age**	0.278 (0.198)	0.164	-0.587 (0.210)	0.006
**Male**	-8.321 (3.986)	0.039	-5.119 (4.476)	0.256
**Ex-smoker**	-15.657 (4.752)	0.001	0.799 (5.104)	0.876
**Current smoker**	-23.222 (14.581)	0.114	-4.183 (15.157)	0.783
**CC16**	0.151 (0.153)	0.326	0.220 (0.166)	0.188
**CCL18**	-0.038 (0.022)	0.087	-0.038 (0.026)	0.140
**CRP**	-0.477 (0.575)	0.408	-0.077 (0.609)	0.900
**IL6**	-0.934 (1.887)	0.622	-1.729 (1.992)	0.388
**IL8**	-0.701 (0.704)	0.322	0.763 (0.737)	0.303
**TNF-alpha**	2.529 (1.941)	0.196	1.390 (2.1<0.00167)	0.523
**SP-D**	<0.001 (<0.001)	0.497	<0.001 (<0.001)	0.564

Table S4. Multivariable model to predict FEV1 or TL_{CO} percentage predicted at baseline in patients with ZZ AATD-COPD

FEV1 or TL_{CO} percentage predicted values were modelled using the equation:
FEV1 or TL_{CO} ~ Age + Sex + Smoking + Biomarkers (CC16 + CCL18 + CRP + IL6 + IL6 + TNF-alpha + SP-D). The beta coefficients (Beta1 and Beta2), standard error (SE1 and SE2), p-values (P1 and P2), adjusted R^2 (R^2) and number included in each model (n) are presented in the table.

	FEV1 (n=110) R^2=0.20		TLCO (n=98) R^2=0.04	
	**Beta1 (SE1)**	**P1**	**Beta2 (SE2)**	**P2**
**Age**	0.315 (0.173)	0.072	-0.503 (0.191)	0.010
**Male**	-7.226 (3.678)	0.052	-2.770 (4.083)	0.499
**Ex-smoker**	-17.050 (4.586)	<0.001	-0.428 (5.035)	0.932
**Current smoker**	-19.552 (14.078)	0.168	-4.319 (14.830)	0.772

Table S5. Multivariable model to predict FEV1 or TL_{CO} percentage predicted at baseline in patients with ZZ AATD-COPD (without biomarkers)

FEV1 or TL_{CO} percentage predicted values were modelled using the equation:

FEV1 or TL_{CO} ~ Age + Sex + Smoking. The beta coefficients (Beta1 and Beta2), standard error (SE1 and SE2), p-values (P1 and P2), adjusted R^2 (R^2) and number included in each model (n) are presented in the table.

	FEV1 (n=94) R^2=0.15		TLCO (n=84) R^2=0.08	
	**Beta1 (SE1)**	**P1**	**Beta2 (SE2)**	**P2**
**Age**	3.167 (0.707)	<0.001	0.007 (0.002)	0.004
**Male**	-4.638 (13.858)	0.739	-0.079 (0.045)	0.082
**Ex-smoker**	43.879 (18.178)	0.018	0.051 (0.058)	0.382
**Current smoker**	18.699 (67.935)	0.784	-0.005 (0.210)	0.981

Table S6. Multivariable model to predict FEV1 and TL_{CO} absolute change in patients with ZZ AATD-COPD (without biomarkers)

FEV1 (mLs/year) or TL_{CO} (mmol/min/kPa/year) absolute change values were modelled using the equation:

FEV1 or TL_{CO} ~ Age + Sex + Smoking. The beta coefficients (Beta1 and Beta2), standard error (SE1 and SE2), p-values (P1 and P2), adjusted R^2 (R^2) and number included in each model (n) are presented in the table.

	FEV1 (n=94) R^2=0.14		TLCO (n=84) R^2=0.13	
	**Beta1 (SE1)**	**P1**	**Beta2 (SE2)**	**P2**
**Age**	0.108 (0.024)	<0.001	0.085 (0.033)	0.011
**Male**	0.541 (0.466)	0.249	-0.007 (0.641)	0.991
**Ex-smoker**	1.205 (0.567)	0.036	0.186 (0.748)	0.804
**Current smoker**	0.343 (2.151)	0.874	-0.477 (2.778)	0.864
**CC16**	-0.021 (0.017)	0.227	-0.053 (0.023)	0.023
**CCL18**	0.001 (0.003)	0.565	-0.005 (0.004)	0.236
**CRP**	-0.017 (0.066)	0.797	-0.086 (0.088)	0.332
**IL6**	0.040 (0.203)	0.843	-0.059 (0.266)	0.826
**IL8**	-0.120 (0.075)	0.114	0.123 (0.099)	0.219
**TNF-alpha**	0.356 (0.228)	0.123	-0.013 (0.304)	0.966
**SP-D**	<0.001 (<0.001)	0.552	<0.001 (<0.001)	0.442

Table S7. Multivariable model to predict FEV1 and TL_{CO} percentage predicted change in patients with ZZ AATD-COPD

FEV1 or TL_{CO} percentage predicted change values were modelled using the equation: FEV1 or TL_{CO} ~ Age + Sex + Smoking + Biomarkers (CC16 + CCL18 + CRP + IL6 + IL6 + TNF-alpha + SP-D). The beta coefficients (Beta1 and Beta2), standard error (SE1 and SE2), p-values (P1 and P2), adjusted R^2 (R^2) and number included in each model (n) are presented in the table.

	FEV1 (n=94) R^2=0.14		TLCO (n=84) R^2=0.02	
	**Beta1 (SE1)**	**P1**	**Beta2 (SE2)**	**P2**
**Age**	0.090 (0.021)	<0.001	0.068 (0.031)	0.029
**Male**	0.270 (0.411)	0.513	-0.482 (0.591)	0.417
**Ex-smoker**	1.115 (0.540)	0.042	0.457 (0.761)	0.550
**Current smoker**	0.200 (2.017)	0.921	-0.412 (2.753)	0.881

Table S8. Multivariable model to predict FEV1 and TL_{CO} percentage predicted change in patients with ZZ AATD-COPD (without biomarkers)

FEV1 or TL_{CO} percentage predicted change values were modelled using the equation: FEV1 or TL_{CO} ~ Age + Sex + Smoking. The beta coefficients (Beta1 and Beta2), standard error (SE1 and SE2), p-values (P1 and P2), adjusted R^2 (R^2) and number included in each model (n) are presented in the table.

Figures

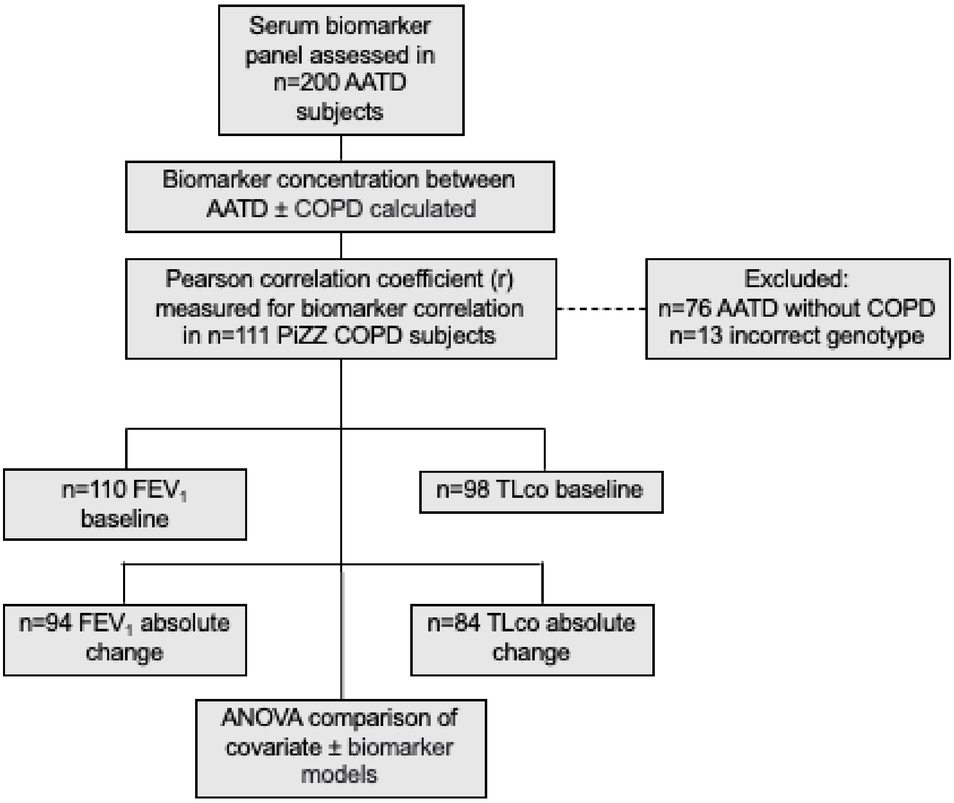

Figure S1: Study flow chart of biomarker measurement and subsequent statistical analyses.

The concentrations of a biomarker panel were measured in n=200 subjects with alpha-1 antitrypsin deficiency. Mann-Whitney U test was performed to calculate significance between the concentrations of biomarkers between AATD subjects with and without COPD. Pearson correlation coefficient (r) was measured only for AATD subjects with COPD and the PiZZ genotype (a total of n=111), of which n=110 subjects had a baseline FEV1 available and n=98 baseline TLco. R was also measured for biomarker correlation with FEV1 absolute change in n=94 subjects and TLco absolute change in n=84 subjects. The difference in model (covariates alone or covariates plus biomarker panel) was assessed using analysis of variance (ANOVA). AATD – alpha-1 antitrypsin deficiency; COPD – chronic obstructive pulmonary disease; FEV1 – forced expiratory volume in 1 second; TLco – transfer capacity of the lung for carbon monoxide.

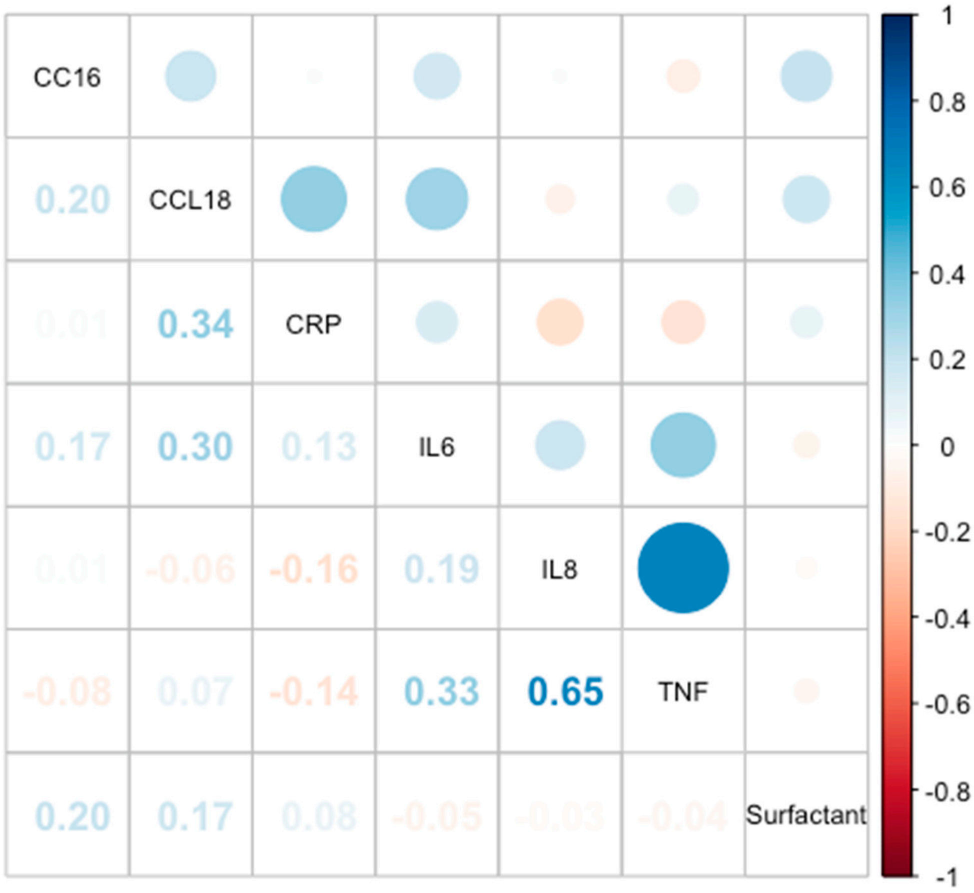

Figure S2. Biomarker correlation plot

Biomarker correlation calculated using Pearson correlation coefficient with the coloured scale showing -1 (perfect negative relationship) to 1 (perfect positive relationship)

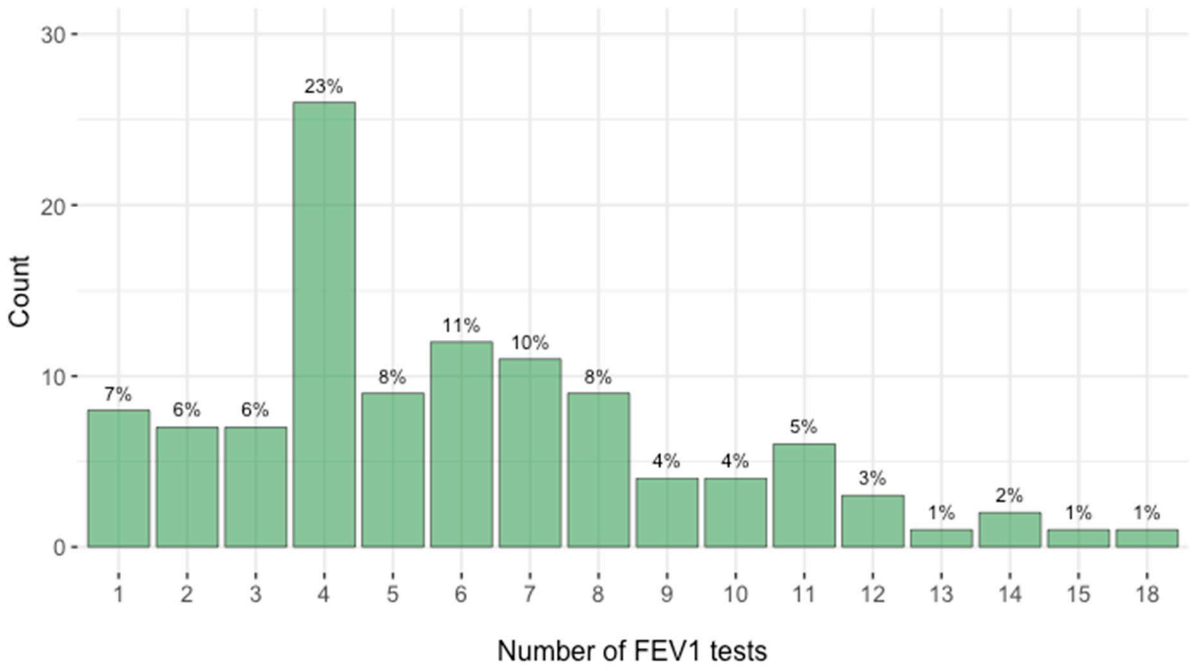

Figure S3. Histogram of number of FEV1 tests per individual

Histogram of the number of FEV1 tests available per individual for ZZ AATD-COPD patients.

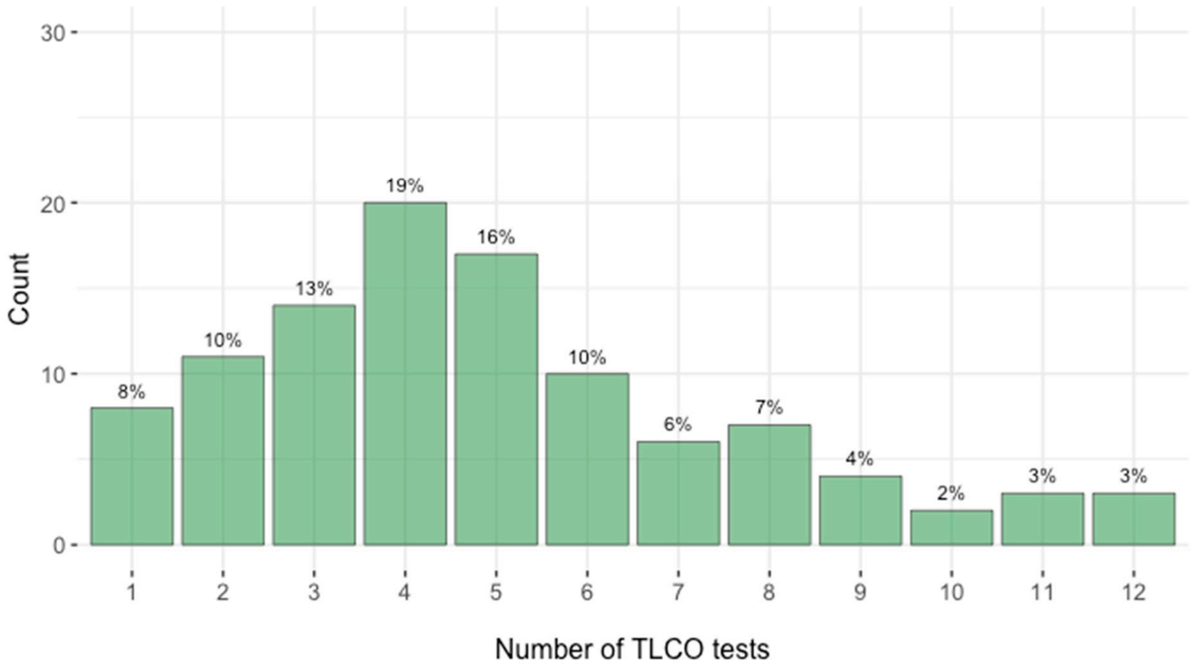

Figure S4. Histogram of number of TLCO tests per individual

Histogram of the number of TLCO tests available per individual for ZZ AATD-COPD patients.

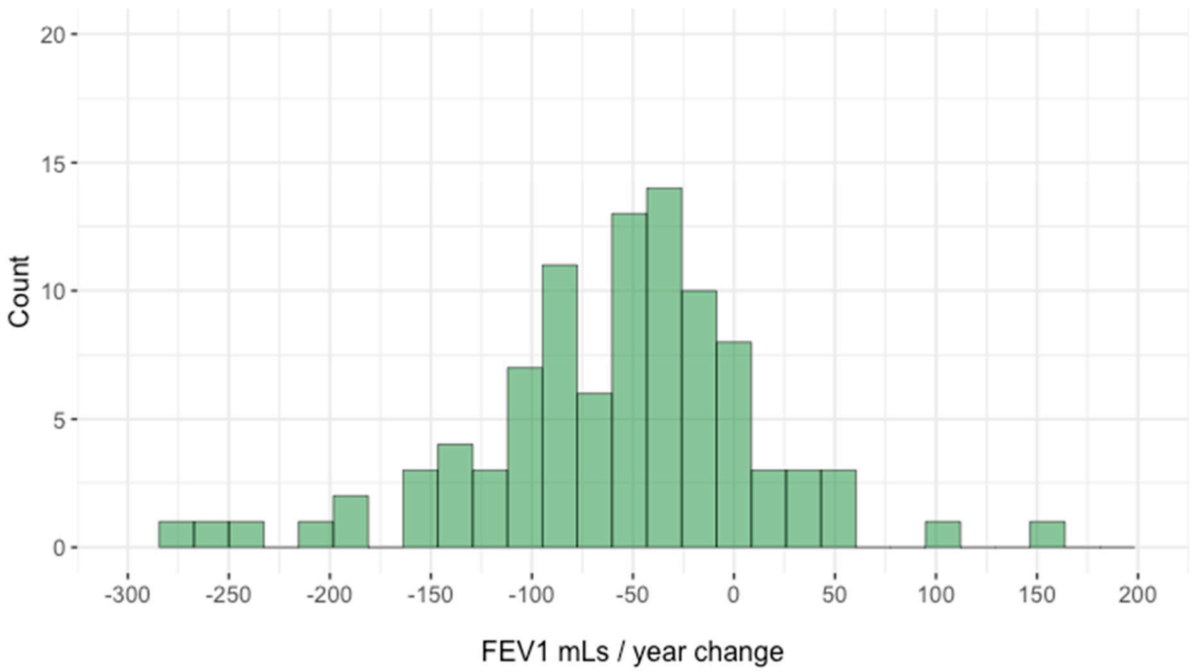

Figure S5. Histogram of FEV1 absolute change.

Histogram of the absolute change in FEV1 (mLs / year) for ZZ AATD-COPD patients.

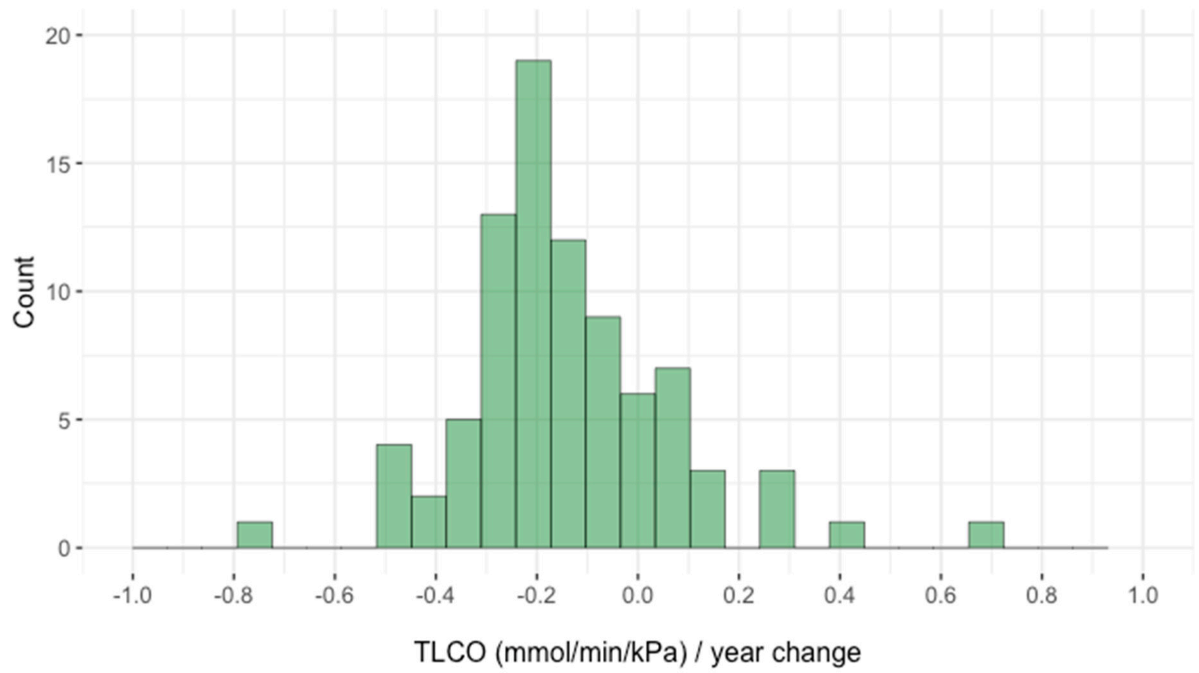

Figure S6. Histogram of TLCO absolute change.

Histogram of the absolute change in TLCO (mmol/min/kPa/year) for ZZ AATD-COPD patients.
